# Surgical Approaches for Stage IVA Thymic Epithelial Tumors

**DOI:** 10.3389/fonc.2013.00332

**Published:** 2014-01-14

**Authors:** Mark Shapiro, Robert J. Korst

**Affiliations:** ^1^The Daniel and Gloria Blumenthal Cancer Center, Paramus, NJ, USA; ^2^The Division of Thoracic Surgery, Department of Surgery, The Valley Hospital, Valley Health System, Ridgewood, NJ, USA

**Keywords:** stage IVA thymoma, neoadjuvant therapy, surgery, metastasectomy, extrapleural pneumonectomy, hyperthermic intrathoracic chemotherapy

## Abstract

Thymic epithelial tumors (TET) are rare mediastinal neoplasms that can metastasize to the pleural space (stage IVA). Complete surgical resection remains the backbone of therapy for patients with early stage TET, however, the role of surgery in the management of patients with stage IVA disease is not fully defined. Published reports in this regard are mainly small, retrospective, and uncontrolled, with unclear inclusion criteria. Surgical options to manage pleural disease include metastasectomy, extrapleural pneumonectomy, and metastasectomy/pleurectomy combined with heated intrapleural chemotherapy. The choice of the most appropriate surgical strategy needs to be individualized according to the quantity and location of disease, the patient’s overall condition, as well as operator and institutional expertise. In the majority of cases, metastasectomy of pleural implants will be sufficient to achieve a complete resection. The available literature suggests that in selected patients with stage IVA TET, delivery of neoadjuvant chemotherapy followed by complete resection is a viable treatment option that can be associated with long-term survival.

## Introduction

Thymic epithelial tumors (TET) are rare neoplasms, but they are the most frequent anterior mediastinal tumors in adults. According to SEER (Surveillance, Epidemiology, and End Results) data, the overall incidence of TET is 0.13 per 100,000 person-years (1). Histologically, TET are biphasic tumors, comprised of epithelial and lymphocytic components. The World Health Organization system uses epithelial cellular atypia as well as the ratio of epithelial cells to lymphocytes to classify TET into types A, AB, B1, B2, B3, and carcinoma. TET are also classified using tumor stage. The staging system that has gained worldwide acceptance is the one proposed by Masaoka et al. (2) and subsequently modified by Koga (3). Stage IVA TET is defined as disease which has spread to either the pleural space or the pericardium but is not contiguous with the primary tumor in the mediastinum. Although only approximately 7% of patients with TET will initially present with stage IVA disease (4), the pleural space represents the most frequent site of recurrence following resection (5).

## The Rationale for Surgical Intervention in Stage IVA TET

Surgical resection represents the mainstay of treatment for early stage TET. The vast majority of patients with early stage (Masaoka stage I and II) disease are cured by surgical resection alone. For patients with stage III TET, the most consistent prognostic factor that has emerged is the ability to perform a complete surgical resection with negative margins (R0 resection) (6–8). Based on the importance of complete resection in patients with stage III TET, a surgical approach has also been advocated for patients with stage IVA disease as well, despite the absence of any published controlled clinical trials. It is well accepted that surgical resection for stage IVA disease appears to be safe. Many stage IVA patients present in middle age and are otherwise healthy without significant comorbid risk factors. As a result, the majority of published studies that describe surgical resection for stage IVA TET report no operative mortality (9–12).

The impact of surgical resection for stage IVA TET on oncologic outcome is less clear. Yano and colleagues (9) reported the outcomes of 21 patients with stage IVA thymoma who underwent multimodality treatment including neoadjuvant corticosteroid therapy followed by surgical resection and adjuvant radiation therapy. Seven additional patients did not undergo attempted resection. Patients that proceeded with surgical resection had 73.1% 5 year overall survival versus 0% for patients who did not. The patients who were able to undergo complete macroscopic resection (*n* = 15) enjoyed a longer recurrence-free survival than those with subtotal resection (*n* = 6) with a 3-year disease-free rate of 63.6% compared to 20.8%, respectively (*p* = 0.009). There was no mortality at 30 days, and perioperative morbidity occurred in four (19%) patients. In another study of 23 patients with invasive thymoma published by Froudarakis and colleagues (13), the authors analyzed outcomes based on two treatment paradigms: surgical resection ± chemo(radio)therapy (*n* = 11), or radiotherapy ± chemotherapy (*n* = 12). Patients in the operative group experienced longer overall survival than those in the non-operative group (*p* < 0.0001).

The importance of complete resection has also been reported in patients with stage IVA TET. In this regard, Rena and colleagues (14) analyzed the outcomes of 18 patients with Stage IVA thymoma, and demonstrated that outcome was poor in patients with incomplete resection. After a mean followup period of 82 months, disease specific 10 year survival was 52% following complete macroscopic resection compared to 0% after incomplete resection (*p* = 0.048). Similar outcomes were reported by Regnard and colleagues (7) after mean of 8 years of followup. For patients with stage IVA TET who underwent R0 resection (*n* = 12), the 5-year overall survival was 64%, compared to 34% for the patients (*n* = 6) who were incompletely resected.

In summary, surgical resection for stage IVA TET appears to be safe, with very low operative mortality reported. Although some studies report enhanced survival after R0 resection relative to patients who do not undergo resection, these reports are small, mainly retrospective, and subject to selection bias. Without controlled clinical trials, it remains unclear if the enhanced oncologic outcome achieved with surgery is due to the resection itself, or merely a reflection of probable lower tumor burden in the patients selected for a surgical approach.

## Surgical Approaches for Patients with Stage IVA Thymic Tumors

For patients with stage IVA TET, surgical options to manage the pleural disease include metastasectomy, extrapleural pneumonectomy (EPP), and metastasectomy/pleurectomy combined with heated intrapleural chemotherapy. The choice of the most appropriate surgical strategy needs to be individualized according to the quantity and location of disease, as well as operator and institutional expertise. In many situations, the surgeon does not know the extent of resection that will need to be performed until the chest is explored. No prospective clinical trials have been published that examine differences in outcome according to the surgical approach.

### Metastasectomy

The most common surgical approach for TET that has metastasized to the pleura is localized excision of all visible intrathoracic disease (metastasectomy). When patients initially present with stage IVA disease radiographically and the primary tumor has not yet been resected, the incision must be planned in order to accommodate exploration of the hemithorax containing the pleural disease. In most circumstances, a median sternotomy alone is not adequate for pleural exploration, and oftentimes the combination of a partial median sternotomy with an anterior thoracotomy (hemiclamshell) facilitates exposure of the pleural cavity. In the uncommon circumstance where both pleural cavities need to be explored, bilateral anterior thoracotomies combined with a transverse sternotomy (clamshell) may be required for exposure.

Since the pleural space is the most common site of recurrence following initial resection of TET, it is not uncommon to also address pleural disease in the absence of a mediastinal lesion. In these cases, the pleural space is best approached using a standard, posterolateral thoracotomy. This incision permits visualization of the entire hemithorax, allowing the surgeon adequate exposure to employ a wide variety of resection techniques. Although video assisted thoracic surgery (VATS) has gained acceptance for the management of other intrathoracic disease states, including early stage TET (15, 16), its use for advanced lesions is limited due to the need to thoroughly explore the entire pleural cavity and resect multiple lesions in many cases.

Once the chest has been explored, the surgeon tailors the operation according to the number, size, and location of the metastatic lesions. Deposits that are confined to the parietal pleura are usually easily resected with a margin of normal pleura. Visceral pleural deposits can either be removed using electrocautery or, if larger, by performing a wedge resection of the lung with an automatic stapler. In the unusual circumstance where a large visceral pleural lesion is invading the lung, an anatomic pulmonary resection (e.g., lobectomy) may be required. Reported results with this approach show perioperative mortality of 0% and morbidity up to 39% (9–12, 14).

### Extrapleural pneumonectomy

Occasionally, the surgeon may explore the chest of a patient with the intent of performing metastasectomy, only to find extensive, diffuse visceral, and parietal pleural implants which are too numerous to remove individually. In this circumstance, a decision must be made regarding the performance of EPP. EPP involves resection of the entire lung in addition to its surrounding pleural envelope, thereby removing all pleural tissue. This operation is performed through a posterolateral thoracotomy, and involves resection and reconstruction of the hemidiaphragm as well. Usually considered as a “last resort,” EPP has been reported to be associated with long-term survival in patients with stage IVA TET (17–19). A potential benefit of the performance of EPP is that higher doses of postoperative radiotherapy can be administered to the involved hemithorax with less toxicity than if the lung remained *in situ* (20, 21).

The largest series of EPP for thymic tumors was published by Fabre and colleagues (17). The authors analyzed the outcome of 17 patients that underwent EPP for Masaoka stage IVA thymoma over a period of 39 years. Eight patients had recurrent thymoma and nine patients presented *de novo* with stage IVA disease. All patients underwent EPP and an R0 resection was achieved in 11 (65%). Five patients (29.4%) underwent adjuvant therapy consisting of chemotherapy (*n* = 2), radiotherapy (*n* = 1), or both (*n* = 2). With a median followup of 59 months, the 5- and 10-year overall survival was 60 and 30%, respectively. During the followup period, two patients experienced recurrence. Perioperatively, there were no deaths, but the 30-day mortality was 17.6%. In addition, eight patients (47%) experienced major postoperative complications, including four with postpneumonectomy bronchopleural fistula (23%). In another report, Wright and colleagues described five patients who underwent EPP for stage IVA thymoma and demonstrated no operative mortality with a single patient having a major complication. The overall 10 year survival was 50% (18). In summary, radical surgery in the form of EPP represents a “last resort” option for patients with stage IVA TET, but surgical mortality and morbidity are significant and the oncologic benefit is unproven.

### Intraoperative, hyperthermic, intrathoracic chemotherapy

A relatively new surgical approach involves resection of pleural disease with administration of intraoperative hyperthermic, intrathoracic chemotherapy (HITHOC). This strategy has been suggested for stage IVA TET as early as 2001. Using a standard roller pump and heat exchanger, perfusion of the pleural space is initiated without the chemotherapeutic agents at the rate of 1,000–2,000 ml/min with an inflow temperature of 42°C. Once the outflow temperature has stabilized, the chemotherapeutic agent(s) is added and perfusion is performed over a usual period of 60 min.

Refaely and colleagues (22) published the outcome of 15 patients who underwent resection and hyperthermic pleural perfusion with chemotherapy. The procedure included resection with or without pleurectomy, with a solitary patient undergoing EPP. All patients received intraoperative hyperthermic intrapleural cisplatin. During perfusion, the intrapleural temperature reached 40.3–43°C. There was no operative mortality, but complications consisted of significant bleeding (*n* = 2), fever (*n* = 2), and air leak (*n* = 1). Excluding patients with thymic carcinoma (*n* = 5), the 3- and 5-year overall survival rates were 90 and 70%, respectively, with a median followup of 34 months. The authors concluded that surgical resection with HITHOC is safe and seems to offer good local control for patients with stage IVA TET.

This approach has enjoyed a resurgence of interest in recent years (23–26). Several small reports demonstrate no mortality within 30 days, with overall survival rates of 67–89% without evidence of recurrence. However, the length of followup was too short in these studies (18.8–29 months), given that TET may take many years to recur. The largest study was conducted by Yellin and colleagues (26), which evaluated 35 patients. Seventeen patients presented with *de novo* stage IVA disease, 14 with pleural recurrence after a previous resection, while four had thymic carcinoma. There was no systemic toxicity reported with HITHOC, the mortality at 90 days was 2.5%, and major morbidity occurred in four patients (11.4%). The 5- and 10-year overall survival was 81 and 73%, respectively, for *de novo* thymomas, but this decreased to 67 and 56% for patients who presented with recurrent tumors. Patients with thymic carcinoma had the worst outcome, with no patients surviving 5 years. Progression-free survival was higher in patients who were deemed to have undergone an R0 resection compared to incompletely resected patients (*p* < 0.001).

### Photodynamic therapy

The use of the photodynamic therapy (PDT) can potentially add another dimension to treatment of patients with stage IVA TET. This modality has been described in conjunction with radical pleurectomy for patients with pleural mesothelioma (27). However, to date, there are no published reports that evaluated the use of intraoperative PDT for pleural disease in patients with stage IVA TET. Further investigation is needed to see if PDT has a role in treatment of these patients.

## Neoadjuvant Therapy and Stage IVA Thymic Tumors

The use of neoadjuvant chemotherapy followed by surgical resection for thoracic malignancies was initially popularized in patients with locally advanced lung cancer beginning in the 1980s. This concept was then applied to locally advanced TET, with the first study in stage III TET reported in 1991 (28). The rationale behind using neoadjuvant therapy in locally advanced TET patients is that TET are chemoresponsive tumors and the prospect of enhancing resectability using preoperative treatment is attractive, since the ability to perform an R0 resection is a strong prognostic factor. The use of neoadjuvant therapy for patients with stage IVA TET has subsequently emerged as a viable treatment strategy, and has been reported in several, mainly retrospective studies (Table 1).

**Table 1:** **Outcome of patients with stage IVA TET treated with neoadjuvant therapy followed by surgical resection**.

Reference	*n*	Induction response (%)	Complete resection (%)	Median followup (months)	Recurrence rate (%)^b^	5-Year overall survival (%)	10-Year overall survival (%)
Rea et al. (34)^a^	16	100	–	–	–	70^d^	–
Venuta et al. (29)	13	100	76.9	53	10	–	68^e^
Refaely et al. (22)	15	–	66.7	34	30	70	–
Kim et al. (12)^a^	22	77	76	50	–	95	79^f^
Lucchi (10)	11	66.7^a^	72.7	–	–	–	45.7
Lucchi et al. (30)	10	73.3^a^	76.7^a^	94	–	–	76.4
Huang et al. (11)	18	67	67	22	25	78	65
Yano et al. (9)	21	–	71	–	–	73.1	37.6
Cardillo et al. (8)^a^	31	75.6	58.1	77	–	–	57.9
Okereke et al. (31)	15	–	100	57^c^	–	88	50
Rena et al. (14)	18	86	62.5	95^c^	50	85	53

*^a^Includes patients with Masaoka stages III and IVA thymoma*.

*^b^Patients that underwent complete resection*.

*^c^Mean followup*.

*^d^3-Year followup*.

*^e^8-Year followup*.

*^f^7-Year followup*.

Of note, a formal, prospective, phase II clinical trial that evaluated the impact of induction therapy in 22 patients with stage III and IVA thymomas (stage IVA *n* = 10) was published by Kim and colleagues (12). Patients underwent neoadjuvant chemotherapy consisting of cyclophosphamide, doxorubicin, cisplatin, and prednisone, followed by surgical resection, radiation therapy and consolidation chemotherapy. Nineteen of 22 patients completed the planned therapy. In the entire cohort, induction chemotherapy produced a major radiographic response in 17 patients (77%), and 16 patients (76%) were able to achieve an R0 resection. With a median followup of 50.3 months, the overall survival rate was 95% at 5 years and 79% at 7 years. The progression-free survival was 77% at 5 years and 77% at 7 years (12).

As shown in Table 1, the high radiographic response rates to neoadjuvant therapy reflect the chemoresponsiveness of these tumors. In addition, the rate of R0 resection in patients with stage IVA TET following neoadjuvant therapy is in the range of 58–100%. These figures compare favorably to reports of resection without the use of neoadjuvant therapy in patients with stage IVA TET. Studies with surgical resection as a primary therapy followed by adjuvant therapy show the rate of complete resection to be lower (43–63%), with 5 and 10 year overall survival rates in the range of 30–47% (7, 32, 33).

A small number of non-randomized studies have attempted to compare the outcomes of patients with stage IVA TET who undergo treatment with neoadjuvant therapy followed by surgical resection to those who were operated upon primarily. Lucchi and colleagues reported 36 patients with stage III and IVA TET who underwent multimodality treatment with neoadjuvant chemotherapy, surgical resection, and postoperative radiotherapy. This group was compared to 20 patients who were treated by primary surgery and postoperative chemo(radio)therapy (10). In the entire cohort, the radiographic response rate to neoadjuvant chemotherapy was 67%. Stage IVA patients who received neoadjuvant therapy had an R0 resection rate of 72.7% (8 of 11), compared to 40% (2 of 5) in the patients who underwent primary resection. Neoadjuvant therapy also improved survival significantly (*p* < 0.02).

Cardillo and colleagues also compared the use of neoadjuvant therapy followed by surgical resection to primary resection in patients with stage III and stage IVA TET. Unfortunately, the data were not analyzed individually according to tumor stage. After a median followup period of 77 months (8), the 10-year overall survival rate in the group of patients who received neoadjuvant chemotherapy was 57.9%, compared to 38.1% in patients who underwent primary surgical resection. Multivariate analysis confirmed that R0 resection and induction chemotherapy were statistically significant independent predictors of survival.

## Summary

The role of surgical resection in the management of patients with stage IVA TET is not fully defined. Published reports are mainly small, retrospective, and uncontrolled, with unclear inclusion criteria. This makes it difficult to separate any potentially positive effects of surgical intervention from other important variables, including tumor burden. Further, the published literature frequently analyzes stages III and IVA collectively as advanced disease. Despite this, the available literature suggests that in selected patients with stage IVA TET, delivery of neoadjuvant chemotherapy followed by R0 resection is a viable treatment option that is associated with long-term survival. The extent of surgical intervention should be based on the patient’s overall condition and the extent of pleural disease. In the majority of cases, metastasectomy of pleural implants will be sufficient to achieve an R0 resection. In highly selected patients with extensive pleural deposits, EPP remains an option, but significant postoperative morbidity and mortality need to be considered. The role of HITHOC remains undefined, but worthy of further investigation. Given the rarity of stage IVA TET, it will be difficult to answer questions regarding the management of this disease without well-designed, multi-institutional clinical trials in order to achieve significant accrual. The formal organization of physicians interested in TET by the International Thymic Malignancy Interest Group (ITMIG) will help in this regard, allowing clinical trial accrual to be expanded to multiple institutions and countries.

## Author Contributions

Mark Shapiro was involved in the literature search as well as project conception, manuscript writing and final approval. Robert J. Korst was involved in project conception, manuscript writing, critical revisions and final approval.

## Conflict of Interest Statement

The authors declare that the research was conducted in the absence of any commercial or financial relationships that could be construed as a potential conflict of interest.
